# 肺癌住院手术患者临床特征与就诊模式的关系

**DOI:** 10.3779/j.issn.1009-3419.2015.07.10

**Published:** 2015-07-20

**Authors:** 玉田 赖, 龙 田, 骏 樊, 健 黄, 双江 李, 恒 杜, 国卫 车

**Affiliations:** 610041 成都，四川大学华西医院胸外科 Department of Thoracic Surgery, West China Hospital, Sichuan University, Chengdu 610041, China

**Keywords:** 就诊模式, 临床特征, 肺肿瘤, 城市和乡镇, Diagnostic modes, Clinical characteristics, Lung neoplasms, City and Township

## Abstract

**背景与目的:**

肺癌患者的就诊模式(就诊原因)可能决定其治疗方案，而二者之间的关系仍不清楚，研究肺癌住院手术患者的临床特征与就诊模式的关系，有助于临床和卫生管理决策。

**方法:**

分析2013年1月-2013年12月间华西医院胸外科手术治疗的505例肺癌患者临床特征，按照就诊模式分组如下：体检组为无症状或无明显症状下体检发现肺癌入院手术治疗患者(131例)，症状组则为因出现症状而入院诊疗的患者(374例)，分析两组的手术方式、病理分期等方面的差异；同时分析城市、乡镇患者就诊模式方面的不同。

**结果:**

131例体检组患者中，体检的方式以低剂量计算机断层扫描(low-dose computed tomography, LDCT, 46.6%, 61/131)和胸片(51.1%, 67/131)为主，体检发现肺癌入院手术治疗的城市患者(35.4%, 81/229)高于乡镇(18.1%, 50/276)(*P* < 0.001)；体检发现Ⅰ期肺癌患者在城市(46.8%, 59/126)高于乡镇(27.3%, 33/121)(*P*=0.001)。VATS肺叶切除术在体检组(73.3%, 96/131)高于症状组(44.4%, 166/374)(*P* < 0.001)。病理分期为Ⅰ期的肺癌患者在体检组(70.2%, 92/131)显著高于症状组(41.4%, 155/374)(*P* < 0.001)。

**结论:**

城市健康体检人群显著高于乡镇，健康体检有助于发现早期肺癌患者和行微创外科治疗。

## Abstract (en)

**Background and objective:**

Diagnostic modes may play an important role in treatments, but minimal information is available regarding their relationship in patients with lung cancer. This study may contribute to decision making in clinics and public health centers.

**Methods:**

The records of 505 hospitalized surgical patients with lung cancer at the Department of Thoracic Surgery, West China Hospital of Sichuan University from January 2013 to December 2013 were retrospectively reviewed. The patients were categorized into physical examination group (PEG, 131 patients) and symptomatic group (SG, 374 patients). Surgical approach, pathological stage, and diagnostic mode were analyzed.

**Results:**

Low-dose computed tomography (46.6%, 61/131) and computed radiography (51.1%, 67/131) were used as key diagnosis methods in 131 patients in PEG. The percentage of hospitalized surgical patients with lung cancer detected via physical examination in the city (35.4%, 80/229) was also significantly higher than in the township (18.1%, 50/276) (*P* < 0.001). The ratio of stage Ⅰ lung cancer detected via physical examination in the city (46.8%, 59/126) was significantly higher than that in the township (27.3%, 33/121) (*P*=0.001). The proportion of patients who underwent VATS lobectomy was significantly higher in PEG (73.3%, 96/131) than that in SG (44.4%, 166/374) (*P* < 0.001), and the ratio of patients at stage Ⅰ was significantly higher in PEG (70.2%, 92/131) than that in SG (41.4%, 155/374) (*P* < 0.001).

**Conclusion:**

The use of physical examination is more prevalent in cities than that in towns, and its combination with mini-invasive surgical treatment contributes to early diagnosis of patients with lung cancer.

肺癌患者的病理分期不但决定预后，也与手术方式和生活质量有着紧密联系。这些均可能与其就诊模式密切相关。研究^[1-3]^证明低剂量计算机断层扫描(low-dose computed tomography, LDCT)检查可以发现更多的早期肺癌患者，但目前低剂量CT在中国应用于肺癌筛查的比例不高，胸部X线或胸片(computed radiography, CR)仍是较为普遍的肺癌体检或筛查方式，导致大量的早期肺癌患者漏诊，究其原因主要是：一是公众的健康意识不够；二是一些医疗和体检机构仍没有将肺部低剂量CT检查作为服务项目；三是低剂量CT检查没有纳入医保项目之中，导致公众不愿意常规应用LDCT进行肺部检查。同时，国内缺乏关于就诊模式与肺癌患者临床特征及治疗方案的资料，导致科普宣传或卫生管理部门决策中只能采用国外临床数据，而缺乏说服力和公信力。为研究肺癌住院手术患者的临床特征与诊断模式的关系，作者分析了华西医院胸外科2013年肺癌手术和资料完整的患者的临床特征及其就诊模式，具体结果如下。

## 1 资料与方法

### 1.1 临床资料

我们回顾分析2013年1月-2013年12月四川大学华西医院胸外科(本部)手术治疗的616例肺癌患者并对其进行筛选。纳入标准：①病理学检查诊断为原发性肺癌；②手术方式为电视辅助胸腔镜手术(video-assisted thoracic surgery, VATS)或开胸肺叶切除和系统淋巴结清扫术；③临床资料完整。排除标准：①任何肿瘤转移到肺部的转移瘤患者；②病历资料不完整者；③肺癌复发的患者。最终纳入原发性非小细胞癌患者505例，平均年龄为(58.8±10.0)岁。其中男性322例(63.8%)，女性183例(36.2%)；有吸烟史患者254例(50.3%)，无吸烟史患者251例(49.7%)；城市患者229例(45.3%)，乡镇患者276例(54.7%)。根据患者就诊模式不同，分为体检组(25.9%, 131/505)和症状组(74.1%, 374/505)(表 1)。

**1:** 两组患者临床特征 Comparison of clinical characteristics between physical examination group and symptomatic group

	PEG (*n*=131)	SG (*n*=374)	*P*
Age (yr, Mean±SD)	59.6±10.2	58.7±10.1	0.406
Gender			0.044
Male	74 (56.5)	248 (66.3)	
Female	57 (43.5)	126 (33.7)	
Smoking			0.071
Current or ever	57 (43.5)	197 (47.3)	
Never	74 (56.5)	177 (52.7)	
Place of residence			< 0.001
City	81 (61.8)	148 (39.6)	
Township	50 (38.2)	226 (60.4)	
PEG: physical examination group; SG: symptomatic group.			

### 1.2 方法

手术方法：VATS手术方式应用单向式胸腔镜肺叶切除法+系统淋巴结清扫^[4]^。开胸手术方式应用常规后外侧切口，肺叶切除术+系统淋巴结清扫。系统淋巴结清扫左侧必须清扫第5、6、7、8、9、10组淋巴结，右侧包括第2、3、4、7、8、9、10组淋巴结。就诊模式：体检组：包括门诊或各医疗机构应用胸片、LDCT、肿瘤标记物、正电子发射计算机断层显像(positron emission tomography computed tomography, PET-CT)体检的无明显症状的患者。症状组：包括因有临床表现而住院诊疗的患者。术后分期采用国际抗癌联盟(Union for International Cancer Control, UICC)(2009)肺癌分期标准^[5]^。

### 1.3 统计学方法

应用SPSS 19.0软件分析结果，计数资料用实际例数及百分比表示，计数资料间比较采用独立样本的卡方检验或*FISH*检验，计量资料采用均数±标准差(Mean ± SD)表示，采用*t*检验，*P* < 0.05为差异有统计学意义。

## 2 结果

### 2.1 不同就诊模式的资料分析

症状组人群(74.1%, 374/505)临床表现包括咳嗽咳痰(46.7%, 236/505)、疼痛(16.8%, 85/505)、痰中带血或咯血(15.0%, 76/505)以及其他表现(4.2%, 21/505)(图 1)。

**1:**
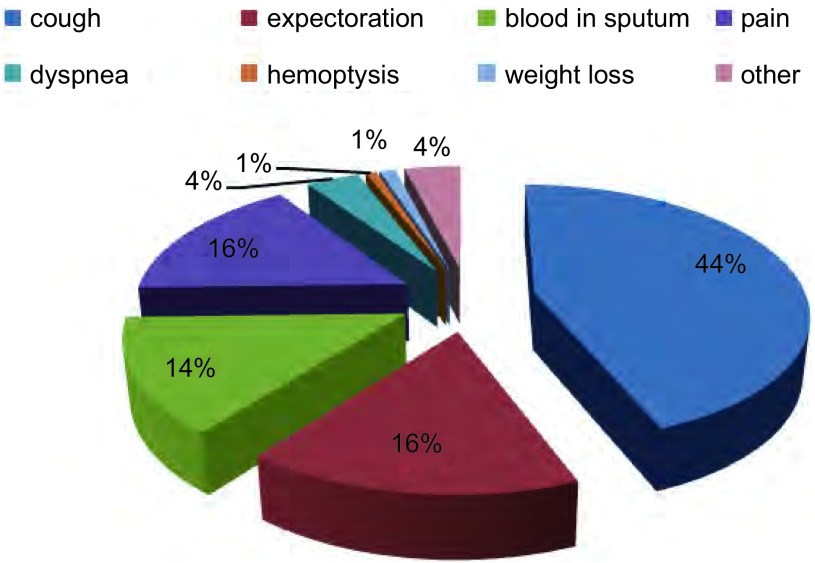
症状组肺癌患者临床症状 Symptoms in symptomatic group

体检组人群(25.9%, 131/505)肺癌检出方式仍以胸片摄影(51.1%, 67 /131)，LDCT(46.6%, 61/131)为主，其他包括肿瘤标记物(1.5%, 2/131)，PET-CT(0.7%, 1/131)(图 2)。其中体检组中，LDCT的应用比例在城市人群(51.9%, 42/81)高于乡镇(38.0%, 19/50)；而胸片在城市(46.9%, 38/81)则低于乡镇(60.0%, 30/50)，差异无统计学意义(*P*=0.113)。

**2:**
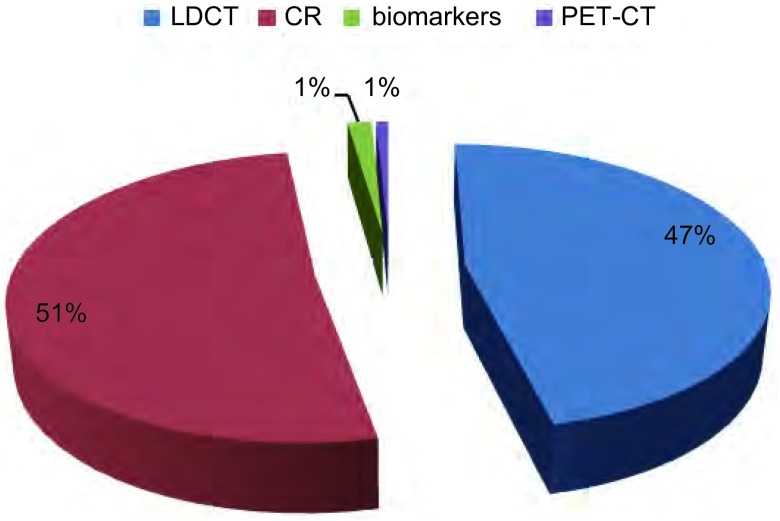
体检组患者检出肺癌方式 Methods for detection in physical examination group; LDCT: low-dose computed tomography; CR: computed radiography; PET-CT: positron emission tomography CT.

### 2.2 城市、乡镇患者就诊模式分析

131例因体检发现肺癌并住院手术治疗的患者中，城市患者比例(61.8%, 81/131)高于乡镇患者(38.2%, 50/131)；城市人群定期体检发现肺癌并住院治疗的患者比例(35.4%, 81/229)高于乡镇(18.1%, 50/276)(*P* < 0.001)。城市Ⅰ期患者中经体检检出肺癌的比例46.8%(59/126)高于乡镇(27.3%, 33/121)(*P*=0.001)(表 2)。

**2:** 城市、乡镇患者的临床特征 Comparison of clinical characteristics between patients in city and patients in township

Characteristic	Patients in city (*n*=229)	Patients in township (*n*=276)	*P*
Age (yr, Mean±SD)	60.9±10.0	57.3±10.0	< 0.001
Gender			0.575
Male	143 (62.4)	179 (64.9)	
Female	86 (37.6)	97 (35.1)
Smoking			< 0.001
Current or ever	94 (41.0)	160 (58.0)	
Never	135 (59.0)	116 (42.0)
Pathological stage			
Stage Ⅰ	126 (55.0)	121 (43.8)	0.012
Stage Ⅱ	48 (21.0)	86 (31.2)	0.010
Stage Ⅲ	46 (20.1)	62 (22.5)	0.517
Stage Ⅳ or tumors metastasizing to lung	9 (3.9)	7 (2.5)	0.373

### 2.3 肺癌住院治疗患者就诊模式与临床特征的关系

262例肺癌住院患者行胸腔镜肺叶切除术(51.9%, 262/505)，243例(48.1%, 243/505)为开胸手术。VATS手术患者比例在体检组(73.3%, 96/131)高于症状组(44.4%, 166/374)；而开胸手术在体检组(26.7%, 35/131)低于症状组(55.6%, 208/374)(*P* < 0.001)。术后病理Ⅰ期肺癌患者在体检组(70.2%, 92/131)高于症状组(41.4%, 155/374)(*P* < 0.001)(表 3)。其中体检组Ⅰ期的肺癌患者中有64.1%(59/92)来自城市，35.9%(33/92)来自乡镇；58.7%(54/92)为经LDCT检出，41.3%(38/92)为经胸片检出。

**3:** 两组肺癌患者病理分期、手术方式选择、住院时间对比 Comparative analysis of pathologic stage, surgical approach, length of stay between PEG and SG

Characteristic	PEG (*n*=131)	SG (*n*=374)	*P*
Pathologic stage (*n*, %)					
Stage Ⅰ	92 (70.2)	155 (41.4)	< 0.001
Stage Ⅱ	19 (14.5)	115 (30.7)	< 0.001
Stage Ⅲ	17 (13.0)	91 (24.3)	0.006
Stage Ⅳ	3 (2.3)	13 (3.5)	0.772
Surgical approach (n, %)			< 0.001
VATS	96 (73.3)	166 (44.4)	
Open	35 (26.7)	208 (55.6)	
Length of stay (d, Mean±SD)					
Total length of stay	16.8±8.6	18.0±8.4	0.162
Preoperative length of stay	8.9±8.0	9.8±7.1	0.203
Postoperative length of stay	7.9±3.7	8.2±5.2	0.610
VATS: video-assisted thoracic surgery.			

## 3 讨论

肺癌的发病率和病死率均居恶性肿瘤之首、且治疗效果相对较差，而早期肺癌的外科治疗却可以达到治愈的目的。因此，通过对健康人群的体检改善肺癌患者的生存率和生活质量，已经成为全社会的焦点^[6, 7]^。

高危人群的每年定期体检对于降低疾病(包括癌症)的发病率和死亡率具有明显效果^[8]^。随着医疗保障制度、医疗服务管理的不断完善和健全，肺癌患者的就诊模式也在不断的变化和发展。尽管国内越来越多的患者是经过定期体检发现肺癌并接受外科诊疗，相较于国外的体检方法和人群范围仍存在明显不足。但是国内关于肺癌的治疗方法和就诊模式的相关研究尚未见报道。我们分析了国内三级甲等医院胸外科通过手术治疗的505例肺癌的临床资料分析表明：有症状就诊模式仍是肺癌患者就诊的主要模式(74.1%)；而无症状或无明显症状下体检发现肺癌入院的131例手术治疗患者中，城市患者体检比例(61.8%)高于乡镇患者(38.2%)。进一步分析发现在城市患体检发现肺癌并接受治疗的人群比例(35.4%)高于乡镇患者(18.1%)。

现有的研究^[1-3]^表明：低剂量螺旋CT检查可以发现更多的早期肺癌患者和降低肺癌患者的死亡率，而胸部X片应用于大规模的筛查肺癌已被证明不能降低肺癌的死亡率，而国内仍有许多医疗机构仍在应用，我们的研究结果也证明胸部X片仍然是最主要的肺癌检出方法(51.1%)，当然同时LDCT也占有重要比例(46.6%)。这可能与胸部X片检查较为经济有关(尤其是对乡镇患者而言)，因此需要进行宣传和提高医生对LDCT发现早期肺癌的认识。

胸腔镜肺叶切除术已成为早期肺癌的标准治疗方法，其无论在手术创伤还是远期生存率均有优势^[9-13]^，尤其是胸腔镜肺癌手术在缩短手术时间和住院时间、降低术中出血、缓解术后疼痛等方面优势更加明显^[14-16]^。也有研究发现胸腔镜肺叶切除术有利于保护心肺功能、改善运动耐力而促进快速恢复并提高肺癌患者术后的生活质量，降低患者术后急性期反应，减轻对患者免疫功能的抑制^[17, 18]^；而定期体检有助于发现更多早期肺癌患者，而使患者选择胸腔镜肺叶切除术的几率更大，从而大大改善肺癌患者的就医体验和生活质量。我们的研究结果也证明了这一点，但是乡镇患者体检率低，也使能行胸腔镜肺叶切除术的患者少，需要我们加强科学宣传和普及。

研究不同就诊模式人群的术后肺癌病理表明：体检组Ⅰ期肺癌患者比例70.2%，高于症状组41.4%，体检组Ⅰ期的肺癌患者中有64.1%(59/92)来自城市，35.9%(33/92)来自乡镇。进一步分析发现城市Ⅰ期患者中经体检检出肺癌的比例46.8%(59/126)高于乡镇(27.3%, 33/121)。这也充分说明了定期体检(肺癌筛查)不但有助于发现早期肺癌，也使患者得到最佳治疗和效果。究其原因总结如下：①各种形式的体检筛查有效地检出了更多的早期肺癌，缩短了从发现到实施诊疗的时间，同时患者可能随后在有关体检筛查部门的建议下继续进行后续的诊疗；②更多早期的无明显相关症状的肺癌可能在体检筛查过程中被发现，避免了患者出现相关症状才就医的情况；③体检组中的城市患者居多，健康意识、经济能力、受教育程度、享有的医疗资源等方面相对优于乡镇患者，一定程度上有利于就医诊疗。现在的事实是LDCT用于肺癌的筛查的比例仍低于胸部X光片，这是需要解决的问题，尤其是医疗工作人员更应该进行宣传并反映在卫生决策中。

综上所述，体检发现肺癌并入院治疗模式下的患者总体上肺癌的临床分期更早，手术方式更倾向胸腔镜肺叶切除方式且治疗效果最佳。加大对定期体检LDCT应用于肺癌筛查的方式的宣传、推广，对肺癌的诊疗有着积极、重要的意义。城市、乡镇患者在体检选择方面的差异提示我们医疗卫生部门更应重视并加强在乡镇地区定期体检的宣传和推广，使更多的居民获益于这种早期肺癌发现、诊断的有效方式。

此次研究并不是前瞻性的随机对照实验，而是回顾性地对患者的病例资料进行分析、评价。同时本研究亦存在一定的不足或缺陷：第一，纳入的505例患者资料均来自于单个研究中心(华西医院本部)；第二，依据排除/纳入标准，我们排除了一些患者，不可避免地对结果造成一定影响；第三，我们希望并需要在以后的研究工作中，进行5年至10年的相关的连续性研究，以此来对体检与诊疗及预后等相关问题展开更全面、深入的探讨。
